# An Ecological Dipole in North American Mast‐Eating Small Mammal Dynamics

**DOI:** 10.1002/ece3.73413

**Published:** 2026-04-29

**Authors:** Jessica H. Barton, Ivy V. Widick, Benjamin Zuckerberg, Courtenay Strong, Marta A. Jarzyna, Jalene M. LaMontagne

**Affiliations:** ^1^ Department of Biology University of Missouri—St. Louis, One University Boulevard St. Louis Missouri USA; ^2^ Department of Forest and Wildlife Ecology University of Wisconsin‐Madison Madison Wisconsin USA; ^3^ Department of Atmospheric Sciences University of Utah Salt Lake City Utah USA; ^4^ Department of Evolution, Ecology and Organismal Biology The Ohio State University Columbus Ohio USA; ^5^ Translational Data Analytics Institute The Ohio State University Columbus Ohio USA; ^6^ Whitney R. Harris World Ecology Center University of Missouri—St. Louis, One University Boulevard St. Louis Missouri USA; ^7^ Center for Conservation and Sustainable Development Missouri Botanical Garden St. Louis Missouri USA

**Keywords:** climate drivers, ecological dipole, macrosystems ecology, NEON, population dynamics, small mammal

## Abstract

Ecological dipoles are the opposite patterns in animal and plant population indices over time in geographically distant areas. Ecological dipoles are recently described macroscale patterns in animal and plant population dynamics, and have been uncovered in mast seeding and in migration patterns of mast‐eating birds at continental scales. It has been predicted that ecological dipoles occur in a range of taxa. We tested for patterns and drivers of spatiotemporal synchrony in the dynamics of hard‐mast eating small mammal populations, along with co‐located climate variables hypothesized to affect small mammal populations directly or indirectly through driving seed availability. We used box‐trapping records from 2013 to 2022 for nine mast‐eating small mammal species from 23 forested National Ecological Observatory Network (NEON) sites in the contiguous United States spanning distances over 4000 km. We found an ecological dipole in small mammal population dynamics, with a decay in synchrony as the distance between pairs of NEON sites increased, with anti‐synchrony in small mammal dynamics at sites separated by > 2000 km. Site proximity, direct climate variables, and indirect climate variables (i.e., time‐lagged responses to mast‐seeding dynamics) were associated with the spatiotemporal synchrony in small mammal abundance. Our study supports the prediction that ecological dipoles may be a generalizable continental‐scale pattern for multiple taxa.

## Graphical abstract

Ecological dipoles are patterns in plant and animal indices of opposite extremes, and many ecological dipoles may exist. We discovered a novel ecological dipole in seed‐eating small mammals in North America using spatial analyses. We also tested for associations between climate variables and small mammal synchrony, and found that site proximity as well as a number of climate variables has influence on small mammal synchrony across time and space.

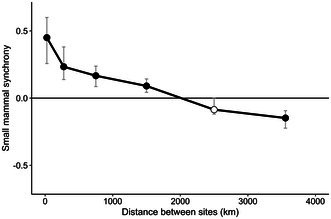

## 1 Introduction

Ecological dipoles are characterized by fluctuations in ecological responses (e.g., population abundance, reproduction) of opposite polarity in populations in geographically distinct areas at a macrosystems scale (Zuckerberg et al. 2020). The concept of ecological dipoles is relatively new, and examples have been reported in spatiotemporal patterns of tree reproduction across the boreal forest of North America (LaMontagne et al. 2020), boreal bird irruptions (Strong et al. 2015), Ponderosa pine post‐fire recruitment (Littlefield et al. 2020), European beech tree reproduction (Vacchiano et al. 2017), and in Mediterranean pine growth (Seim et al. 2015). Climatic dipoles, which are known in atmospheric sciences, can entrain ecological dipoles (Zuckerberg et al. 2020), and affect wide‐scale weather patterns in North America; an example of this is the El Niño‐Southern Oscillation (ENSO) phenomenon, which results in dipoles of cool and wet or hot and dry conditions across North America (McPhaden et al. 2006). Climate dipoles can impact ecological systems; the Indian Ocean Dipole (IOD) can cause decreased breeding success in penguins, since they must dive deeper for food and expend more energy due to increased ocean surface temperatures (Bost et al. 2015). Ecological dipoles may have similar cascading ecological effects, for example, avian species may shift ranges on continent‐level scales due to increased food availability from resource pulses (Strong et al. 2015), changing the ecosystem dynamics of those areas in a given year.

Another group that may have an ecological dipole in their spatiotemporal dynamics across a regional to continental scale is small mammals. At local scales, small mammal population dynamics are influenced by food availability, which affects reproduction and survival (Wolff 1996; Mason‐Romo et al. 2018; Andreassen et al. 2021) and by climate, which has a direct influence on abundance (Batzli 1992; Wolff 1996; Deitloff et al. 2010; Rossi and Leiner 2022) and an indirect influence on abundance through pulsed food availability (Ostfeld et al. 1996; Holland et al. 2015). Mast seeding, the synchronous and highly temporally variable production of seed crops by a population of perennial plants (Kelly 1994; Bogdziewicz et al. 2024), has a considerable effect on small mammals. High seed production increases reproductive success of small mammals which results in an increase in their populations the following year (Ostfeld et al. 1996; Falls et al. 2007; Andreassen et al. 2021), providing a rationale to hypothesize that an ecological dipole may exist in hard mast seed‐eating (or ‘mast‐eating’) small mammal populations. Small mammal population dynamics on a continental scale have yet to be explored, in part due to the field of macrosystems biology being relatively new (Heffernan et al. 2014; Dodds et al. 2021) and the lack of biological datasets of consistent methodology that span broad geographic extents. Seed eating small mammals play key roles as both consumers and prey in forest ecosystems (Ostfeld et al. 1996), and a dipole in their dynamics may have further cascading effects in ecosystems. The National Ecological Observatory Network (NEON), which collects standardized data products at sites across the United States across ecoclimatic domains that represent regions' predominant landscapes (Battelle 2020), is an emerging tool that presents the opportunity to fill this gap in knowledge about small mammal dynamics at a continental scale.

Mast seeding has an ecological dipole in the magnitude of seed produced in North America that is associated with climatic dipoles (LaMontagne et al. 2020). Summer temperatures influence mast seeding dynamics in a variety of species, where the magnitude of masting in the current year is influenced by the difference in the preceding summer temperatures (referred to as ΔT hereafter), not absolute summer temperatures (Kelly et al. 2013, LaMontagne et al. 2021). The climate dipole in summer temperature differentials is the difference in summer temperature between the year prior to seed maturity (e.g., ΔT equals the mean July temperature in year t−1 minus the mean July temperature in year t−2). In temperate regions a higher positive ΔT value corresponds to higher magnitude of seed production, and it has been demonstrated as a predictor of seed production in datasets from multiple families of plants (Kelly et al. 2013; LaMontagne et al. 2021). While there have been efforts to compile mast seeding datasets across regions and globally (Pearse et al. 2017; Hacket‐Pain et al. 2022), the distributions of those data are patchy in time and space, and it is thus challenging to make links directly to where small mammal data have been collected. Due to the connections between masting patterns and climate, climate‐based models have been used as a surrogate for pulsed resources used to predict seed consumer population dynamics (Holland et al. 2015).

In this study, we (i) test for an ecological dipole in mast‐eating small mammal populations across the contiguous United States of America and (ii) determine the role of geographic proximity and climate variability on mast‐eating small mammal synchrony. We analyzed population‐level data on the abundance of nine species of mast‐eating small mammals which have hard mast seeds as a substantial fraction of their diet at 23 forested NEON sites, and the associations of spatiotemporal synchrony in population dynamics with climate data. We hypothesized that (i) there is anti‐synchrony in mast‐eating small mammal population dynamics at large distances between sites, (ii) synchrony in mast‐eating small mammal population dynamics is influenced by synchrony in interannual temperature differentials that are associated with mast seeding, and (iii) a combined effect of synchrony in direct weather variables (current year summer temperatures and winter temperatures) and synchrony in the interannual temperature differential associated with mast seeding will be most influential to spatiotemporal synchrony in mast‐eating small mammal dynamics.

## 2 Materials and Methods

### 2.1 NEON Site Selection

**FIGURE 1:**
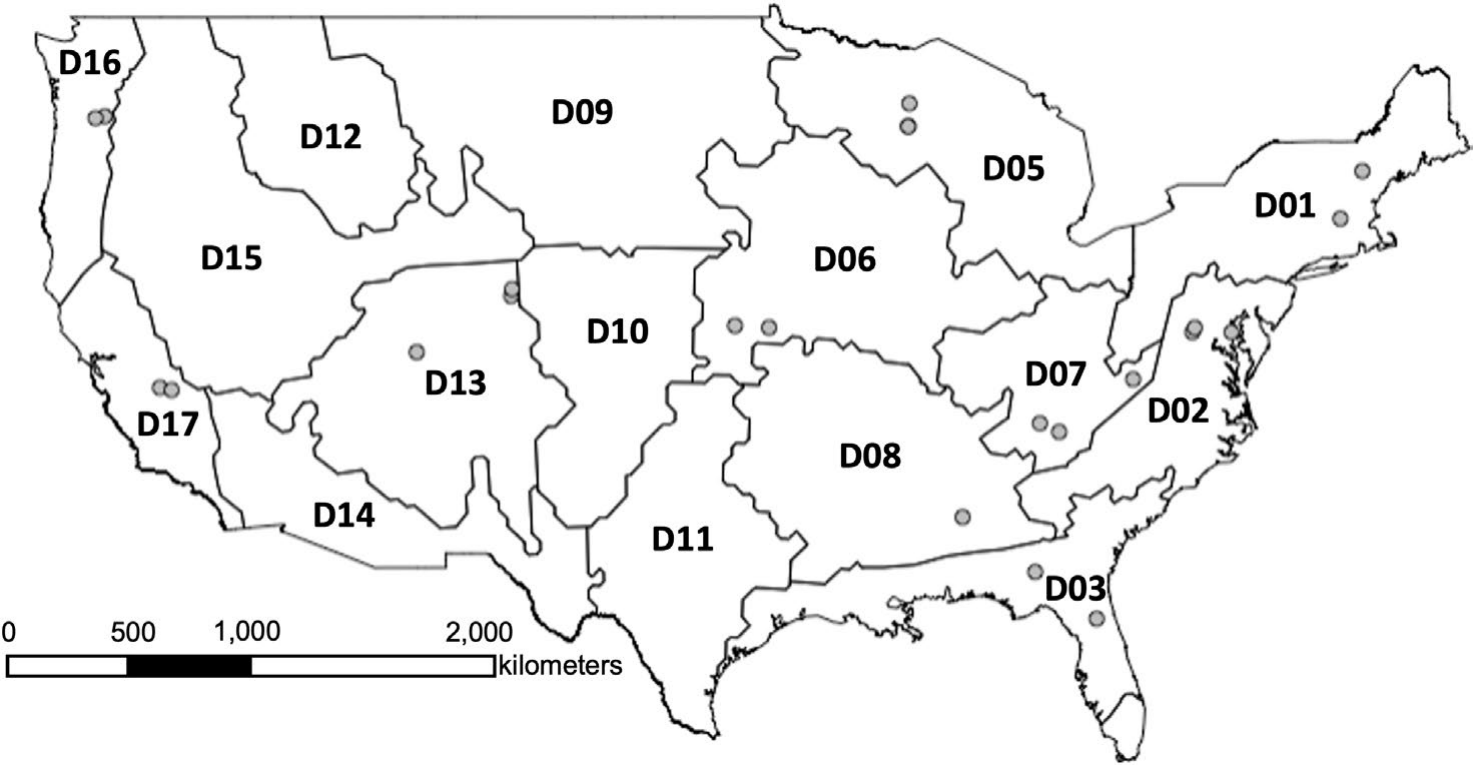
Map of NEON sites used in spatiotemporal analyses. NEON sites are represented by points, overlaid on labeled NEON domains.

We included data from terrestrial forested NEON sites in the contiguous United States with at least one of the following National Land Cover Database (NLCD) land cover classes: ‘Evergreen Forest’, ‘Deciduous Forest’, ‘Mixed Forest’, or ‘Woody Wetlands’; these included sites with mast‐seeding tree species present based on NEON Terrestrial Observation System (TOS) Site Characterization Reports (Table S1). Additionally, we selected sites based on small mammal data quantity, including ≥ 4 years of consecutive data, ≥ 4 months of trapping in a year, and only include captures identified to species (Li et al. 2022). This resulted in using 23 NEON sites, from the state of Florida in the southeast to Washington state in the northwest, with distances between NEON sites ranging from 17 to 4113 km, across 12 ecoclimatic domains (Battelle 2020) (Figure 1, Table S1). See Text S1 for additional rationale on excluding specific NEON sites.

### 2.2 Small Mammal Box‐Trapping Data

We used 10 years of small mammal box‐trapping records from the NEON data portal (NEON 2023) from 2013 to 2022. NEON collects small mammal box‐trapping data, tagging individuals and identifying captured animals to species (Paull et al. 2023; Chesney et al. 2024). Sherman box traps are used to capture animals, and 100 traps are placed in 10 × 10 m grids; the number of grids varies from three to eight depending on the size of the site. The number of grids trapped in a given sampling period is site‐dependent, with factors like site area and other associated logistics influencing the number of grids trapped (Paull et al. 2023, Chesney et al. 2024). At each NEON site, two types of small mammal grids are used; pathogen grids, in addition to typical data collected on trapped small mammals (sex, life stage, etc.) include collection of pathogen samples from small mammals (i.e., blood draws), while diversity grids do not, and only collect typical data on small mammals captured. At least three pathogen grids are designated, and the remaining grids are designated as diversity grids. Pathogen grids are sampled three consecutive nights each sampling bout, while diversity grids are sampled only one night per bout. Each sampling bout consists of three nights of trapping, with four bouts per year being the average for most sites; sampling occurs within 10 days before or after the full moon and does not occur year round at all sites, but is rather centered around the months of peak greenness (Paull et al. 2023, Chesney et al. 2024). In this study, we use both the NEON diversity and pathogen grids (Jarzyna et al. 2022) as we are only interested in the number of individuals and species trapped, which is available from both pathogen and diversity sampling data. The NEON target small mammal species, are defined by NEON as being: (1) non‐volant, (2) nocturnally active, (3) an aboveground forager, and (4) > 5 g and < 500–600 g. The box traps NEON uses are intended to capture smaller mammals, and the target taxa includes cricetids, heteromyids, small sciurids, and introduced murids, and does not include species deemed ‘opportunistic’ such as shrews or large squirrels, even though they may be incidentally trapped (Chesney et al. 2024). We chose species for this study from target species captured at our 23 NEON sites, and based on their “mast‐eating” characteristics, where species were included only if they had an EltonTraits 1.0 ‘seeds’ diet of ≥ 20% (Wilman et al. 2014), in combination with tree seeds being described as part of their diet in field guides (Whitaker Jr. 1988; Reid 2006) and/or scientific studies (Hoffmeister 1951, 1981; Jameson 1952; Merritt and Merritt 1978; Jones and Layne 1993; Genoways et al. 1997; Hanley and Barnard 1999; Morzillo et al. 2003; Elias et al. 2004).

At each NEON site, for each species and year separately, we recorded the total count of unique small mammals captured at both diversity and pathogen grids (Jarzyna et al. 2022). From the NEON site filtering described above, we had a total of 76 site‐species time series. While most NEON sites had a large number of unique individual captures across years, a small number of sites had very few captures, and we removed these from analysis. Specifically, we filtered out datasets with very low captures (i.e., maximum of one individual for at least 2 years) for the raw number of small mammals captured in a given time series, this resulted in a total of 70 site‐species time series for our analysis. We additionally recorded the total yearly trap nights to account for sampling effort (based on the nights spent trapping and traps placed per night) because the nights spent trapping varied by site and year, and number of traps placed varied by night. For each species, we then calculated the yearly unique small mammal individuals captured per trap night per year from both types of grids at each site as our small mammal index, and have defined this species‐level index as ‘populations’ (Micheli et al. 1999). Since the index was calculated separately for each species, the total number of small mammal indices (populations) corresponds to the number of species‐site combinations included in our analysis. While NEON's small mammal data support capture–recapture modeling, its high computational demands were beyond the scope of this study, and our focus was not on interspecific comparisons but on estimating within‐species population trends over time. Indeed, raw capture counts have been shown to effectively track single‐species temporal dynamics (Slade and Blair 2000; Hopkins and Kennedy 2004; Schwemm et al. 2018).

We ultimately included a total of nine mast‐eating small mammal species: *Myodes gapperi* , *Neotoma floridana* , *Ochrotomys nuttalli* , *Peromyscus boylii* , *Peromyscus leucopus* , *Peromyscus keeni* , *Peromyscus maniculatus* , *Peromyscus truei* , and *Podomys floridanus* (see Table S2 for species list and diets). While many of the species included are generalist consumers, consuming a wide variety of available food items in their environments, they are also opportunistic consumers and respond to pulses of seeds in the environment due to mast seeding (Conrod and Reitsma 2015). Community composition varied slightly among sites, with the number of species present at each site between *n* = 1 and *n* = 3. Some species occurred only at a few sites (e.g., *N. floridana* at four sites), while others were found at many sites (e.g., *P. maniculatus* at 19 sites); one species is unique to one site ( *P. floridanus* ), many sites (21 out of 23) have a species from the *Peromyscus* genus present, and two sites only have one species present (BLAN and JERC), though these species are present at four and 13 other sites, respectively.

### 2.3 Climate Data

We downloaded Daymet meteorological data (2009–2022) for each NEON site using the R package ‘daymetr’ (Hufkens et al. 2018; Thornton et al. 2022). We included climate variables in the analysis that reflected summer conditions (mean July temperature, total June precipitation, ΔT of mean July temperature for year t−2 minus year t−3 (ΔT_2‐3_)), and winter conditions (mean January temperature, total January precipitation; Table 1). The temperature differential ΔT_2‐3_ was used because small mammals in year *t* are influenced by mast seeding in year t−1, and mast seeding in year t−1 is influenced by the difference in the two preceding July temperatures, year t−2—year t−3. We use climate data to approximate mast seeding's influence (Holland et al. 2015), as NEON does not collect mast seeding data. In temperate regions, tree species vary in their seed‐development time, being either 2 or 3 years (LaMontagne et al. 2024). Of all tree species at our selected sites, based on NEON vegetation structure surveys from the past 12 years (NEON 2025), 79% of tree species have a two‐year seed development time (bud differentiation in year 1 and pollination and seed maturity in year 2), and 21% of tree species have a three‐year seed development time between bud differentiation and seed maturity. Thus, the ΔT_2‐3_ temperature differential reflects the majority of tree species at the NEON sites. As detailed below, our statistical modeling considered climate variability that operated directly on small mammals, or indirectly through links to cues for mast seeding (Figure S3; Holland et al. 2015).

**TABLE 1:** Climate variables included in analyses. Temp_t_ is the mean daily temperature for the month, and Precip_t_ is the total precipitation (rain + snow equivalent) for the month. ΔT_2‐3_ is the temperature differential of 2 and 3 years prior to year. *T*_3‐4_.

Climate variable	Description	Reason for inclusion	References
**Direct variables**			
JulyTemp_t_	Mean July temperature of the current year	July temperature is used in previous mast seeding ecological dipole work; July temperature has been shown to have a positive influence on small mammal abundance	LaMontagne et al. (2020), Polyakov et al. (2021)
JunePrecip_t_	Total June precipitation of the current year	June precipitation has been shown to have a positive effect on small mammal abundance	Mason‐Romo et al. (2018), Polyakov et al. (2021)
JanuaryTemp_t_	Mean January temperature of the current year	January temperature is the coldest average month in the United States, used as a proxy for the harshness of winter	NOAA (2022)
JanuaryPrecip_t_	Total January precipitation of the current year	January precipitation has been used in previous small mammal population abundance studies as a winter climate variable	Scarlett (2004), Deitloff et al. (2010)
**Indirect variables**			
ΔT_2‐3_	Difference in mean July temperature between 2 and 3 years prior to the current year	Temperature differentials are good at predicting mast seeding, which influences small mammal populations	Kelly et al. (2013), Meyer and Pendleton (2015), Holland et al. (2015), Vacchiano et al. (2017), Krebs et al. (2017), LaMontagne et al. (2021)

### 2.4 Analysis

We used spatial correlogram analysis to quantify patterns of spatiotemporal synchrony (Koenig and Knops 1998) in mast‐eating small mammal dynamics, which can detect ecological dipoles when used over large spatial extents (LaMontagne et al. 2020). We conducted Spearman correlations between pairs of sites for time series of annual population sizes, with a time series for each species at a NEON site. We established distance classes for spatial correlograms across the 4113 km between sites of: < 50 km, ≥ 50 km to < 500 km, ≥ 500 km to < 1000 km, ≥ 1000 km to < 2000 km, ≥ 2000 km to < 3000 km and ≥ 3000 km. Significance for each distance class was tested comparing the median correlation value to zero using bootstrapping percentile confidence intervals with *n* = 1000 permutations using the ‘boot’ package in R (Canty and Ripley 2017; Thulin 2022). Spatiotemporal patterns in five climate variables were also analyzed with spatial correlograms for summer (JulyTemp_t_, JunePrecip_t_, ΔT_2‐3_) and winter (JanuaryTemp_t_, JanuaryPrecip_t_) using the same distance classes as described above.

To determine what drives small mammal synchrony at NEON sites, we performed multiple regression on distance matrices (MRM) analysis on small mammal populations at NEON sites (Goslee and Urban 2007), which is an extension of partial Mantel analysis (Lichstein 2007). The dataset used in MRM analysis retained time‐series of individual species at a site, and were not combined or averaged to create a single time‐series for each site prior to calculation of small mammal spatial correlation matrices (LaMontagne et al. 2024). A climate variable spatial correlation matrix was also calculated. We used the MRM function in the ‘ecodist’ package to determine what influences small mammal synchrony (Goslee and Urban 2007; LaMontagne et al. 2024), with the synchrony in mast‐eating small mammal population abundance over time between NEON sites as the response variable and the synchrony in climate variables and proximity between sites as predictor variables. MRM operates on distance matrices, and because each site appears in multiple pairwise comparisons, the data are non‐independent by design. Due to the constraints of MRM analysis, we were unable to account for low trapping effort at sites, but accounted for low trapping effort by using effort in our small mammal index, eliminating sites with low overall effort, and standardizing our data. We evaluated four models following the methodology of Koenig et al. (2017) and LaMontagne et al. (2020): (1) A space‐only model including proximity as the predictor variable, (2) a direct climate variable model including JulyTemp_t_, JunePrecip_t_, JanuaryTemp_t_, and JanuaryPrecip_t_, (3) an indirect climate variable model including ΔT_2‐3_ and (4) a saturated model using all variables. Statistical significance was tested performing *n* = 1000 permutations of each model. We chose these climate variables to include in the analysis following tests of multicollinearity: we began with a list of multiple ΔT and July temperature variables (e.g., ΔT_1‐2_, July temperature in year t−1, respectively) and, of those highly correlated, only chose one variable based on the biological significance. We chose ΔT_2‐3_ because its timeframe most influences tree seed production in year t−1 (in relation to year *t* of small mammals), and current July temperature due to hot summer temperatures being influential on small mammal populations. The primary unit of analyses is synchrony of small mammals at NEON sites, and MRM analysis examined small mammal synchrony and synchrony of associated response variables. All analyses were conducted in R version 4.0.3 (R Core Team 2022).

## 3 Results

### 3.1 Spatial Synchrony

**FIGURE 2:**
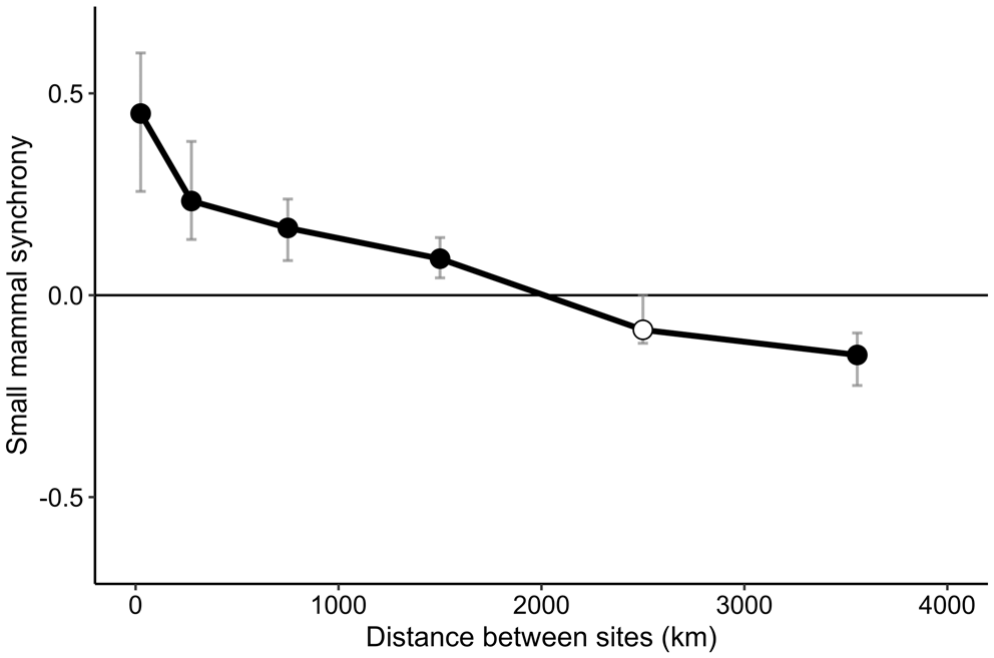
Spatial correlogram for populations of box‐trapped mast‐eating small mammals across the contiguous United States. Points along the line are located at the midpoint of each distance bin and indicate the median correlation in the bin; filled black points are significantly different from 0 while unfilled white points are not significantly different from 0. Significance for spatial correlograms was tested using bootstrapped 95% confidence intervals with *n* = 1000 permutations (gray lines). Note that the bootstrapped confidence intervals are not necessarily symmetric because they aren't constrained by a symmetric parametric distribution.

Spatial correlogram analysis revealed an ecological dipole in the synchrony of mast‐eating small mammal population dynamics (Figure 2). At sites that were the closest together, the median synchrony value for small mammal populations was the greatest, synchrony declined with distance, and at distances greater than 2000 km, median synchrony in mast‐eating small mammal population dynamics was significantly negative (anti‐synchronous) between NEON sites. Note that if the distance between NEON sites were ignored, the overall synchrony in small mammal dynamics between all pairs of sites was close to zero (mean = 0.10), while synchrony in small mammal dynamics within NEON sites was shifted to be positive (mean = 0.37; Figure S1a). The closest NEON sites to each other were 16 km (NIWO and RMNP), 27 km (BLAN and SCBI), and 30 km (ABBY and WREF) apart, and summing all the species present for each year, correlations in small mammal dynamics over time between these pairs of sites were 0.94, 0.45, and 0.71, respectively. Conversely, the furthest NEON sites from each other were 4113 km (SJER and BART), 4079 km (SOAP and BART), and 4059 km (SJER and HARV) apart, with correlations in small mammal dynamics over time of 0, −0.20, and −0.79.

**FIGURE 3:**
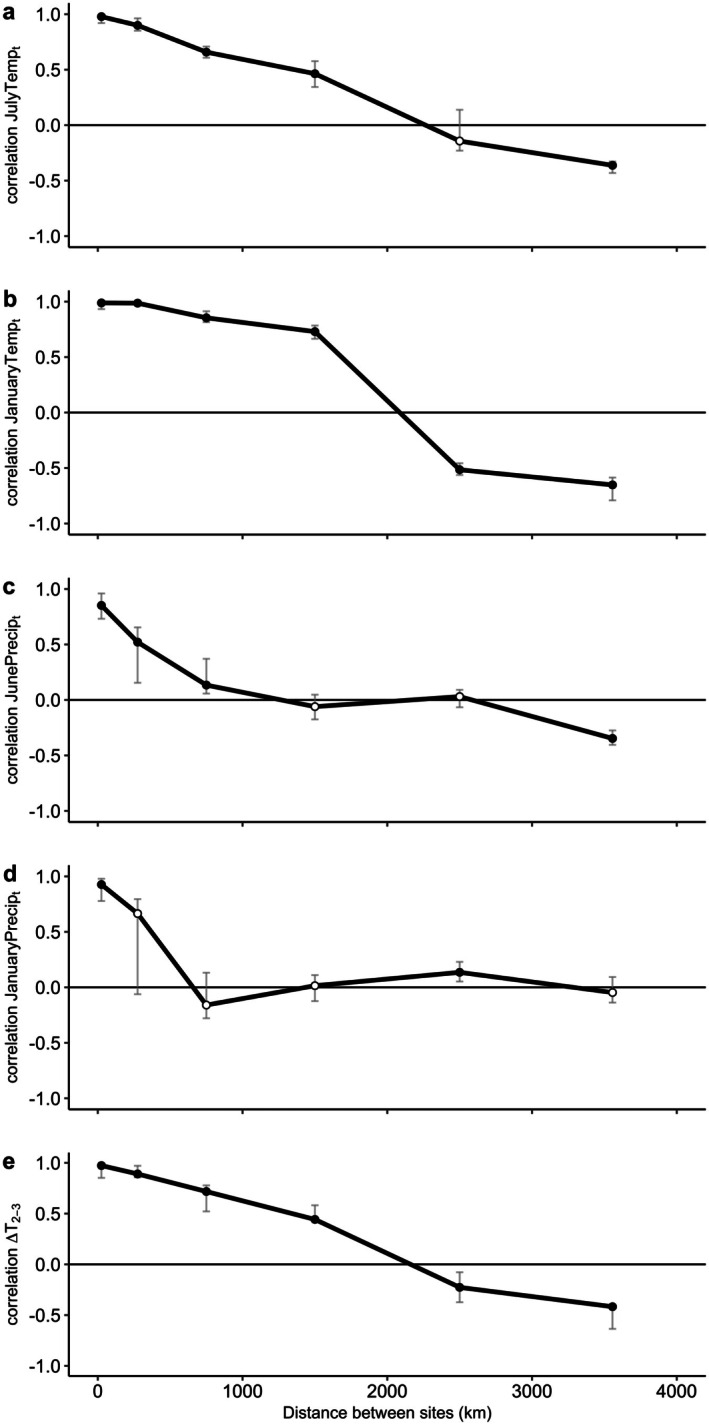
Spatial correlograms for synchrony in climate variables (a) JulyTemp_t_, (b) JanuaryTemp_t_, (c) JunePrecip_t_, (d) JanuaryPrecip_t_, and (e) ΔT_2‐3_ at NEON sites across the contiguous United States, 2013–2022. Points along the line represent the median of each distance bin; filled black points are significantly different from 0 while unfilled white points are not significantly different from 0. Significance for spatial correlograms was tested using bootstrapped 95% confidence intervals with *n* = 1000 permutations (gray lines). Note that the bootstrapped confidence intervals are not necessarily symmetric because they aren't constrained by a symmetric parametric distribution.

Spatial correlogram analysis on climate variables showed high local synchrony that generally declined with increased distance between NEON sites. For JulyTemp_t_, median synchrony between sites was significantly negative at distances > 2500 km (Figure 3a), and in JanuaryTemp_t_, synchrony became significantly negative at > 2000 km (Figure 3b), with JanuaryTemp_t_ having a sharper decline to negative correlations than JulyTemp_t_. Synchrony in JunePrecip_t_ and JanuaryPrecip_t_ between sites was significantly positive when close together, then remained around zero in mid‐distance classes, and JunePrecip_t_ was significantly negatively correlated among sites > 3000 km apart (Figure 3c,d). The ΔT_2‐3_ correlogram showed a downward pattern of synchrony as distance between sites increased (Figure 3e), with significant negative synchrony between sites > 3000 km apart.

### 3.2 Small Mammal Dynamics and Climate Drivers

All four of the MRM analysis models for synchrony in small mammal population dynamics at NEON sites as the response variable, and proximity and climate variable synchrony as the predictor variables were significant (Table 2). Proximity (*p* = 0.001) in the space‐only model was significantly positively associated with synchrony in mammal counts, indicating closer NEON sites being more highly synchronous. In the direct climate model, synchrony in JulyTemp_t_ (*p* = 0.001), JanuaryTemp_t_ (*p* = 0.001), and JunePrecip_t_ (*p* = 0.004) was significantly positively associated with greater synchrony in mast‐eating small mammal dynamics. In the indirect climate model, ΔT_2‐3_ was significant (*p* = 0.001). Proximity was also significant in the saturated MRM model (*p* = 0.001), as well as JulyTemp_t_ (*p* = 0.001) and JunePrecip_t_ (*p* = 0.029). Figure S3 shows the associations between the spatiotemporal synchrony in mast‐eating small mammal population dynamics at NEON sites and each of the climate variables included in MRM analysis.

**TABLE 2:** MRM results for small mammal populations at NEON sites with spatial, direct climate, and indirect climate models using total yearly unique small mammals per trap night as the response variable.

Model & Variables	Coefficient	*p* (variable)	*p* (model)
**1. Space**			**0.001**
**Proximity**	**0.086**	**0.001**	
**2. Direct climate**			**0.001**
**JulyTemp**_**t**_	**0.059**	**0.001**	
**JunePrecip**_**t**_	**0.019**	**0.005**	
**JanuaryTemp**_**t**_	**0.023**	**0.001**	
JanuaryPrecip_t_	0.008	0.219	
**3. Indirect climate**			**0.001**
**ΔT**_**2‐3**_	**0.067**	**0.001**	
**4. Saturated model**			**0.001**
**Proximity**	**0.067**	**0.001**	
**JulyTemp**_**t**_	**0.036**	**0.001**	
**JunePrecip**_**t**_	**0.013**	**0.036**	
JanuaryTemp_t_	−0.012	0.078	
JanuaryPrecip_t_	0.004	0.527	
ΔT_2‐3_	0.006	0.498	

*Note:* Statistical significance was tested performing *n* = 1000 permutations of each model. Bolded values indicate significance of the MRM model itself, or significance of variables within the MRM model.

## 4 Discussion

In this study, we report a previously unknown ecological dipole in North American mast‐eating small mammals using 10 years of standardized small mammal box‐trapping data from NEON. We found spatiotemporal synchrony in small mammal populations at sites closer than 2000 km and anti‐synchrony in small mammal populations at sites > 2000 km apart. This anti‐synchrony is representative of an ecological dipole. Regional synchrony in small mammal populations at scales of up to 1000 km has been reported (Bowman et al. 2008); our work provides additional evidence for this local and regional synchrony of small mammal populations and reports novel anti‐synchrony in mast‐eating small mammal dynamics at a continental scale.

We found that spatial synchrony of mast‐eating small mammals was influenced by a number of climate variables, and beyond proximity, mean July temperature in the current year and June precipitation in the current year were significant in every MRM model they were included in. Proximity was expected to be significant due to the Moran effect, where variability in environmental conditions can synchronize animal and plant populations (Moran 1953; Ranta et al. 1997). Previous studies have also shown that environmental variation and animal and plant populations are more similar the closer they are geographically (Liebhold et al. 2004). At this large of a geographical scale, the Moran effect (as opposed to other phenomena, such as dispersal) is likely the cause of the wide‐scale synchrony we observed. The small mammal synchrony correlogram, and the climate correlograms, all exhibited high localized synchrony, and a range of low to no to anti‐synchrony at the highest distance classes. The precipitation correlograms generally began with high local correlation, declined, and then hovered around zero, with no notable negative trends downwards indicating a dipole; this was expected for precipitation correlograms, which have shown a similar pattern in previous dipole work (LaMontagne et al. 2020). For NEON sites that are geographically close together (16–30 km apart), the level of correlations in small mammal dynamics was highly positive, but varied; this could be due to the differing types of land cover represented at these sites. For example, SCBI and BLAN (27 km apart) had a lower than expected correlation of 0.45, but SCBI primarily consists of deciduous mature and young secondary forest, while BLAN is a mosaic of landscape types, consisting of several land types found in urban–rural landscapes, including forests, grasslands, and pastures (Krauss 2018a). Conversely, NIWO and RMNP (16 km apart) and ABBY and WREF (30 km apart) have similar landscape types, with all sites sharing a dominant NLCD class of evergreen forest (Krauss 2018b, 2018c, 2018d), and had high correlations between sites (0.94, 0.71, respectively).

We hypothesized that synchrony in lagged mean July temperature, combined with synchrony in direct climate drivers, would be most influential to small mammal synchrony, as a combined effect of current year drivers and resource availability could have the potential to be most influential for mast‐eating small mammals versus any of these variables alone. JanuaryTemp_t_ was significant only in the direct climate model, not the saturated model. Harsh winter weather can decrease overwinter survival in small mammal populations (Korslund and Steen 2006), but NEON box‐trapping data are collected primarily in the spring and summer months, where an effect of JanuaryTemp_t_ may not be well reflected due to the time period between data collection and the month of January. Summer temperature also influences small mammal populations (Deitloff et al. 2010), with higher summer temperatures being associated with higher recruitment and survival (Polyakov et al. 2021) and extreme summer temperatures being negatively associated with small mammal survival (Lewellen and Vessey 1998). Synchrony in JulyTemp_t_ was significant in all models it was included in, potentially demonstrating the influence of summer temperatures in the current year having immediate effects on small mammal populations. Synchrony in the temperature differential ΔT_2‐3_ of mean July temperatures was only significant in the indirect climate model. We expected ΔT_2‐3_ to be significant in all models it was included in, and it being significant only in the indirect climate model and not the saturated model suggests that there may be some kind of indirect effect of climate variability on small mammal synchrony in North America through mast seeding, though other factors are likely to influence small mammal populations as well. With mast seeding, ΔT influences seed production directly, while ΔT works indirectly on small mammals, so associations may not be as strong. Small mammals also consume a wide variety of food; if no mast seeding material is available for mast‐eating small mammals, they resort to other food sources. This could be another reason ΔT is not as influential as we hypothesized. Though ΔT_2‐3_ is not significant in all models it is included in, synchrony in ΔT_2‐3_ is highly correlated with synchrony in JulyTemp_t_ (0.95), suggesting that these variables are highly related, and since we found that JulyTemp_t_ is highly influential through MRM analysis, ΔT_2‐3_ may be just as influential. The ΔT model is a predictor of mast abundance for a variety of masting species, with a higher value of ΔT generally being associated with higher seed production (Kelly et al. 2013; Holland and James 2015; LaMontagne et al. 2021; Bogdziewicz et al. 2024) and has been used to approximate mast seeding's influence on rodent outbreaks in forested ecosystems when mast seeding data are not available for that area (Holland et al. 2015). Differentials in mean July temperature work well as predictors of mast seeding for many species in North America (LaMontagne et al. 2013, 2020, 2021), and because mast seeding data are not available from NEON sites, we elected to use temperature differentials in lieu of them. Note that masting forecasting from weather data is not perfect, for example, due to the potential influence of drought (Wion et al. 2025), and differing masting forecasting models may be appropriate for differing geographical areas (Journé et al. 2023).

During mast years, small mammal species primarily eat the hard mast seed that is in abundance for food, and otherwise can switch to other sources of food as many small mammals are generalists (Ostfeld and Keesing 2000); but, all small mammals in this study do not consume the same hard mast seeds (Table S2), and this variation in diet could affect the levels of synchrony between species within NEON sites and the variation seen across years in the total number of mast‐eating small mammals across all species (Figure S2). Additionally, the trait differences in hard mast seeds consumed (i.e., seed development rates of 2‐ vs. 3‐year tree species), could contribute further to this; if one species consumes a primarily 3‐year tree species' seeds, they may not be synchronous with other species at the same site, responding to different time‐lagged variables. Nearly 80% of tree species at our NEON sites had a 2 year development time, and when co‐occurring 2‐ and 3‐year tree species are analyzed for synchrony in seed production, more synchrony appears among these species than by chance, and though traits do play a role in synchrony, this pattern overall holds (LaMontagne et al. 2024).

Ecological dipoles are broadly influenced by climate dipoles, such as the El Niño‐Southern Oscillation (ENSO) phenomenon, which results in dipoles of cool and wet or hot and dry conditions across North America (McPhaden et al. 2006), and the Indian Ocean Dipole (IOD) which produces warm ocean temperatures in the Indian Ocean, influencing temperature dipoles across Australia (Zuckerberg et al. 2020). Climate modes—like ENSO and the North Atlantic Oscillation—often act as environmental “bridges,” synchronizing mast seeding (mass, intermittent seed production) across regions and link short term weather triggers to evolutionary strategies (Ascoli et al. 2021). Because these broader climate patterns have predictable periodicities and spatial coherence, they both cue and reinforce interannual and decadal variability in plant reproduction, aligning proximate mechanisms (e.g., resource cues, weather vetoes) with fitness‐enhancing outcomes like predator satiation and efficient pollination (Ascoli et al. 2021). Such teleconnections can also drive lagged ecological processes that interact synergistically with reproductive cycles, creating adaptive benefits on both ecological and evolutionary scales. Many mast seeding tree species have been linked to these modes of climate variability in several continents (Ascoli et al. 2021), however, species, and potentially location, specific patterns have been found in response to modes of climate variability, where different species in the same location may respond to ENSO with increased cone reproduction while another may not (Wion et al. 2021). This could be particularly relevant to our study, where small mammals have differing diets, and may consume seeds of trees that are sensitive, or insensitive to, modes of climate variability, and therefore may not be affected by certain modes of climate variability.

Climate patterns are shifting over time due to climate change, including North American precipitation dipole patterns, changes in the origin and magnitude of ENSO patterns, and shifts in the position and strength of the arctic polar vortex (Zuckerberg et al. 2020; Bai et al. 2024). This begs the question of the implications of climate change on ecological dipoles and whether they will also shift as average temperatures become higher over time. In the mast seeding ΔT model, the difference in temperature between the two previous years—not the magnitude—is what drives mast seeding, so mast seeding may be insensitive to higher temperatures and climate change (Kelly et al. 2013). Small mammal dipoles, if primarily influenced by ΔT and masting, may potentially be the same, though small mammals are also vulnerable to climate change, with negative impacts from higher temperatures over time predicted (Morueta‐Holme et al. 2010; Santoro et al. 2017; de Castro Evaldt et al. 2024). Long‐term work on small mammal ecological dipoles will be needed to test whether small mammal dipoles shift over time, and NEON is planned to run for 30 years (Keller et al. 2008).

Future studies could follow our discovery of the North American small mammal ecological dipole to explore other potential ecological dipoles across trophic levels that may be affected by small mammals having a dipole, such as predators and parasites like ticks. These potential ecological dipoles, however, could be influenced by a number of environmental factors and would likely not be directly related to small mammal populations alone. Future studies could also collect data to test for the direct influence of mast seeding on small mammal populations. Widespread mast seeding data collected would enhance conclusions on the influence of mast seeding dynamics on mast‐eating small mammal populations on a continental scale. Another step after this study is to uncover the drivers of small mammal abundance at NEON sites as other factors, aside from mast seeding, such as predation or climate alone, can influence small mammal physiology and survival (Kausrud et al. 2008; Krebs et al. 2017), and independently drive small mammal populations. We used multiple regression on distance matrices analysis, and while it measured synchrony, which we were interested in, it did not allow us to test for interactions or random effects, or potentially look at the interaction between current year drivers and prior year cues. We explored linear models with this dataset to determine what climatic variables were driving the abundance of mast‐eating small mammals at NEON sites across and within our 12 NEON domains in a synthetic analysis, but no variables included were clear drivers of mast‐eating small mammal abundance, likely due to the nature of NEON domains as they fit into distinct eco‐climatic regions with unique ecological and climatological conditions (e.g., varying greatly in mean annual temperatures and precipitation levels; results not reported). Therefore a more nuanced, site‐by‐site, or domain specific approach examining the drivers of small mammal abundance at NEON sites would have to be taken, and that was not aligned with our goals of testing for the continent‐wide patterns of mast‐eating small mammal dynamics and associated climate drivers at that scale. Climate can have cascading effects down trophic levels—it influences mast seeding, which then influences small mammal populations, bird populations, and other trophic levels (Zuckerberg et al. 2020, Bai et al. 2024), but these dynamics may be anti‐synchronous at a macrosystems scale, where certain eco‐climatic domains could have differing responses to climate and drivers such as temperature.

Here, we examined mast‐eating small mammal synchrony at local to continental scales, uncovered an ecological dipole in mast‐eating small mammal populations across North America, and the potential climate drivers of synchrony in mast‐eating small mammal populations across a large geographic scale. Climate teleconnections have the potential to synchronize environmental conditions over multiple scales, creating simultaneous resource pulses that cascade through ecosystems by fueling species interactions, influencing population cycles, pollination dynamics, seed predation, and even fire regimes. They essentially structure when and where these pulses occur, making them key drivers of community‐level patterns and evolutionary dynamics at local, regional, and continental scales. The opportunity to explore mast‐eating small mammal populations on a continental scale has been limited until the early 2000s (Heffernan et al. 2014), with efforts on widening and standardizing long term ecological data collection becoming more prevalent recently. NEON employs common sampling protocols at all sites, and thus NEON data provides the opportunity to study unexplored ecological dynamics on broad scales (Battelle 2020). We predict that further ecological dipoles will be discovered using this analytical framework.

## Author Contributions

**Jessica H. Barton:** formal analysis (lead), investigation (lead), writing – original draft (lead), writing – review and editing (lead). **Ivy V. Widick:** methodology (supporting), writing – review and editing (supporting). **Benjamin Zuckerberg:** conceptualization (equal), funding acquisition (equal), methodology (equal), writing – review and editing (equal). **Courtenay Strong:** funding acquisition (equal), methodology (equal), writing – review and editing (equal). **Marta A. Jarzyna:** methodology (supporting), writing – review and editing (equal). **Jalene M. LaMontagne:** conceptualization (equal), funding acquisition (equal), methodology (equal), writing – review and editing (equal).

## Funding

This work was supported by the University of Wisconsin‐Madison, Romnes Faculty Fellowship; University of Missouri‐St. Louis, Desmond Lee Endowed Professorship; National Science Foundation, DEB‐1926221, DEB‐1926341, DEB‐1926428, DEB‐2525033.

## Conflicts of Interest

The authors declare no conflicts of interest.

## Supporting information


**Figure S1:** Frequency distribution of small mammal synchrony in annual unique small mammals/trapnight for a) small mammal populations within the same NEON site (at distance class = 0 km; mean = 0.37, median = 0.35 *n* = 21 sites with multiple species) and b) small mammal populations across sites regardless of distance (mean = 0.10, median = 0.10).
**Figure S2:** Temporal patterns of seed‐eating small mammal populations within NEON domains, 2013–2022. Each line represents the standardized total yearly number of small mammals/trapnight for populations at each NEON site (standardized between 0 and 100), organized by domain. The line types (solid, dashed, or dash‐dotted) represent different NEON sites within a given domain. Domains are separated geographically into roughly ‘Western’ or ‘Eastern’ for comparisons between near and far sites. Note that some data from 2020 is missing due to COVID‐19 related constraints on staffing and field sampling. Values for yearly unique mammals per trap night were standardized between 0 and 100 across years prior to analysis to facilitate comparisons between sites and species with different baseline population sizes.
**Figure S3:** Relationships between the synchrony of mast‐eating small mammal population dynamics and variables used in MRM analysis. Note that these figures represent direct associations between the response and predictor variables and show coefficients and relationships based on individual MRM models; these could vary from the partial MRM coefficients for these variables in the overall MRM analysis. Regression lines shown are for significant variables, and saturated MRM coefficients in the bottom right corner. Terms in the MRM included site proximity (ranging from 0 to 1, with 0 being the most distant NEON sites) and pairwise synchrony for a suite of climate variables. Each symbol represents a pair of species. Regression lines are shown with 95% confidence intervals.
**Table S1:** NEON site names and locations included in analysis. Coordinates are latitude and longitude in decimal degrees, and references are for NEON TOS Site Characterization Reports which report the presence of plant and tree species within the NEON site boundaries.
**Table S2:** Mast‐eating small mammal species used in analyses (*n* = 9).
**Text S1:** Rationale for exclusion of specific NEON sites and notes on site‐specific missing data. CLBJ was excluded due to high mean July temperatures when compared to other sites (ranging from 26.8°C to 31.9°C). Through personal research of the site and conferment with NEON ecologists from Domain 13, pinyon pine, a masting tree species, is rare at ONAQ, and may not have a substantial influence on the small mammal populations there, so was excluded. The sites YELL and TEAK, while having collected more than four years of data, do not have at least four years of data for our target small mammal species, and were excluded. The site DELA was eliminated based on data quality, even though it met inclusion criteria, as it only had a record for one of our nine species, with count values of one in each year. Data from 2020 are unavailable at the TALL, MOAB, SERC, and SOAP field sites in our dataset due to the COVID‐19 pandemic that limited the ability of field crews to conduct small mammal trapping; similarly, data are unavailable for YELL in 2022, which otherwise would have been included in our dataset, after the northern Yellowstone area experienced a historical flooding event.

## Data Availability

Datasets used for this research are as follows: small mammal box‐trapping data from 2013 to 2022 are publicly available from the NEON database (NEON 2023; https://doi.org/10.48443/nvpj‐4j94), and daily surface climatological data from 2009 to 2022 are publicly available from the Daymet database (Thornton et al. 2022; https://doi.org/10.3334/ORNLDAAC/2129). A derived mammal index dataset was used in analysis for this study. This mammal dataset, associated metadata, and the R code used for analyses are stored in Dryad: https://doi.org/10.5061/dryad.tdz08kqbm.
