# Supplementary material for: An Ecological Dipole in North American Mast‐Eating Small Mammal Dynamics
Source: Ecol Evol. 2026 Apr 29;16(5):e73413. doi: 10.1002/ece3.73413 (PMC13126089; doi:10.1002/ece3.73413)
Supplement: Supplementary file 1 — Figure S1: Frequency distribution of small mammal synchrony in annual unique small mammals/trapnight for a) small mammal populations within the same NEON site (at distance class = 0 km; mean = 0.37, median = 0.35 n = 21 sites with multiple species) and b) small mammal populations across sites regardless of distance (mean = 0.10, median = 0.10). Figure S2: Temporal patterns of seed‐eating small mammal populations within NEON domains, 2013–2022. Each line represents the standardized total yearly number of small mammals/trapnight for populations at each NEON site (standardized between 0 and 100), organized by domain. The line types (solid, dashed, or dash‐dotted) represent different NEON sites within a given domain. Domains are separated geographically into roughly ‘Western’ or ‘Eastern’ for comparisons between near and far sites. Note that some data from 2020 is missing due to COVID‐19 related constraints on staffing and field sampling. Values for yearly unique mammals per trap night were standardized between 0 and 100 across years prior to analysis to facilitate comparisons between sites and species with different baseline population sizes. Figure S3: Relationships between the synchrony of mast‐eating small mammal population dynamics and variables used in MRM analysis. Note that these figures represent direct associations between the response and predictor variables and show coefficients and relationships based on individual MRM models; these could vary from the partial MRM coefficients for these variables in the overall MRM analysis. Regression lines shown are for significant variables, and saturated MRM coefficients in the bottom right corner. Terms in the MRM included site proximity (ranging from 0 to 1, with 0 being the most distant NEON sites) and pairwise synchrony for a suite of climate variables. Each symbol represents a pair of species. Regression lines are shown with 95% confidence intervals. Table S1: NEON site names and locations included in analysis. Coo [file ECE3-16-e73413-s001.docx]

**Appendix**

**Text A1.** Rationale for exclusion of specific NEON sites and notes on site-specific missing data.

CLBJ was excluded due to high mean July temperatures when compared to other sites (ranging from 26.8°C - 31.9°C). Through personal research of the site and conferment with NEON ecologists from Domain 13, pinyon pine, a masting tree species, is rare at ONAQ, and may not have a substantial influence on the small mammal populations there, so was excluded. The sites YELL and TEAK, while having collected more than four years of data, do not have at least four years of data for our target small mammal species, and were excluded. The site DELA was eliminated based on data quality, even though it met inclusion criteria, as it only had a record for one of our nine species, with count values of one in each year. Data from 2020 are unavailable at the TALL, MOAB, SERC, and SOAP field sites in our dataset due to the COVID-19 pandemic that limited the ability of field crews to conduct small mammal trapping; similarly, data are unavailable for YELL in 2022, which otherwise would have been included in our dataset, after the northern Yellowstone area experienced a historical flooding event.

**Table A1.** NEON site names and locations included in analysis. Coordinates are latitude and longitude in decimal degrees, and references are for NEON TOS Site Characterization Reports which report the presence of plant and tree species within the NEON site boundaries.

| **Site ID** | **Name** | **Location** | **Domain** | **Coordinates** | **TOS Site Characterization Report** |
| --- | --- | --- | --- | --- | --- |
| ABBY | Abby Road | Washington, USA | Pacific Northwest (D16) | 45.762439,  -122.33032 | (Krauss 2018a) |
| BART | Bartlett Experimental Forest | New Hampshire, USA | Northeast (D01) | 44.06389, -71.28737 | (Krauss 2018b) |
| BLAN | Blandy Experimental Farm | Virginia, USA | Mid-Atlantic (D02) | 39.033698,  -78.041788 | (Krauss 2018c) |
| GRSM | Great Smoky Mountains National Park | Tennessee, USA | Appalachians & Cumberland Plateau (D07) | 35.68896, -83.50195 | (Krauss 2018d) |
| HARV | Harvard Forest | Massachusetts, USA | Northeast (D01) | 42.53691, -72.17265 | (Krauss 2018b) |
| JERC | The Jones Center at Ichauway | Georgia, USA | Southeast (D03) | 31.194839,  -84.468623 | (Krauss 2018e) |
| KONZ | Konza Prairie Biological Station | Kansas, USA | Prairie Peninsula (D06) | 39.10077, -96.56307 | (Krauss 2018f) |
| MOAB | Moab | Utah, USA | Southern Rockies & Colorado Plateau (D13) | 38.24828, -109.38827 | (Krauss 2018g) |
| MLBS | Mountain Lake Biological Station | Virginia, USA | Appalachians & Cumberland Plateau (D07) | 37.378314,  -80.524847 | (Krauss 2018d) |
| NIWO | Niwot Ridge | Colorado, USA | Southern Rockies & Colorado Plateau (D13) | 40.05425, -105.58237 | (Krauss 2018g) |
| ORNL | Oak Ridge National Laboratory | Tennessee, USA | Appalachians & Cumberland Plateau (D07) | 35.964128,  -84.282588 | (Krauss 2018d) |
| OSBS | Ordway-Swisher Biological Station | Florida, USA | Southeast (D03) | 29.68928, -81.99343 | (Krauss 2018e) |
| RMNP | Rocky Mountain National Park | Colorado, USA | Central Plains (D10) | 40.27590, -105.54596 | (Krauss 2018h) |
| SCBI | Smithsonian Conservation Biology Institute | Virginia, USA | Mid-Atlantic (D02) | 38.89292, -78.13949 | (Krauss 2018c) |
| SERC | Smithsonian Environmental Research Center | Maryland, USA | Mid-Atlantic (D02) | 38.89013, -76.56001 | (Krauss 2018c) |
| SOAP | Soaproot Saddle | California, USA | Pacific Southwest (D17) | 37.03337,  -119.26219 | (Krauss 2018i) |
| SJER | San Joaquin Experimental Range | California, USA | Pacific Southwest (D17) | 37.10878, -119.73228 | (Krauss 2018i) |
| STEI | Steigerwaldt-Chequamegon | Wisconsin, USA | Great Lakes (D05) | 45.50894, -89.58637 | (Krauss 2018j) |
| TALL | Talladega National Forest | Alabama, USA | Ozarks Complex (D08) | 32.95047, -87.39326 | (Krauss 2018k) |
| TREE | Treehaven | Wisconsin, USA | Great Lakes (D05) | 45.49369, -89.58571 | (Krauss 2018j) |
| UKFS | University of Kansas Field Station | Kansas, USA | Prairie Peninsula (D06) | 39.04043, -95.19215 | (Krauss 2018f) |
| UNDE | University of Notre Dame Environmental Research Center | Wisconsin, USA | Great Lakes (D05) | 46.23391, -89.53725 | (Krauss 2018j) |
| WREF | Wind River Experimental Forest | Washington, USA | Pacific Northwest (D16) | 45.82049, -121.95191 | (Krauss 2018a) |

**Table A2**. Mast-eating small mammal species used in analyses (n = 9).

| **Scientific Name** | **Common Name** | **Seed Diet** | **Reference** |
| --- | --- | --- | --- |
| *Myodes gapperi* | Southern red-backed vole | Nuts*, conifer seeds | Merritt and Merritt 1978 |
| *Neotoma floridana* | Eastern woodrat | Hardwood seeds, acorns | Genoways et al. 1997 |
| *Ochrotomys nuttalli* | Golden mouse | Acorns | Morzillo et al. 2003 |
| *Peromyscus boylii* | Brush mouse | Acorns, conifer seeds | Jameson Jr. 1952 |
| *Peromyscus keeni* | Northwestern deer mouse | Conifer seeds | Hanley and Bernard 1999 |
| *Peromyscus leucopus* | White-footed mouse | Acorns | Elias et al. 2004 |
| *Peromyscus maniculatus* | Deer mouse | Acorns, conifer seeds | Jameson Jr. 1952 |
| *Peromyscus truei* | Piñon mouse | Acorns, piñon nuts | Hoffmeister 1951; Hoffmeister 1981 |
| *Podomys floridanus* | Florida mouse | Acorns | Jones and Layne 1993 |
|  |  |  |  |

*Nuts are defined by the US Forest Service as dry, single-seeded fruits with a high oil content (e.g. acorns, hickory nuts, walnuts, pecans; (U.S. Forest Service 2021).


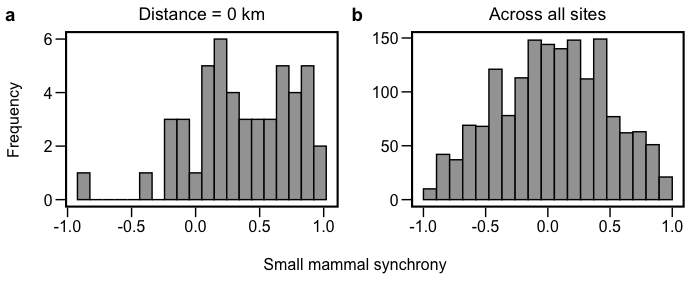


**Figure A1.** Frequency distribution of small mammal synchrony in annual unique small mammals/trapnight for a) small mammal populations within the same NEON site (at distance class = 0 km; mean = 0.37, median = 0.35 n= 21 sites with multiple species) and b) small mammal populations across sites regardless of distance (mean = 0.10, median = 0.10).


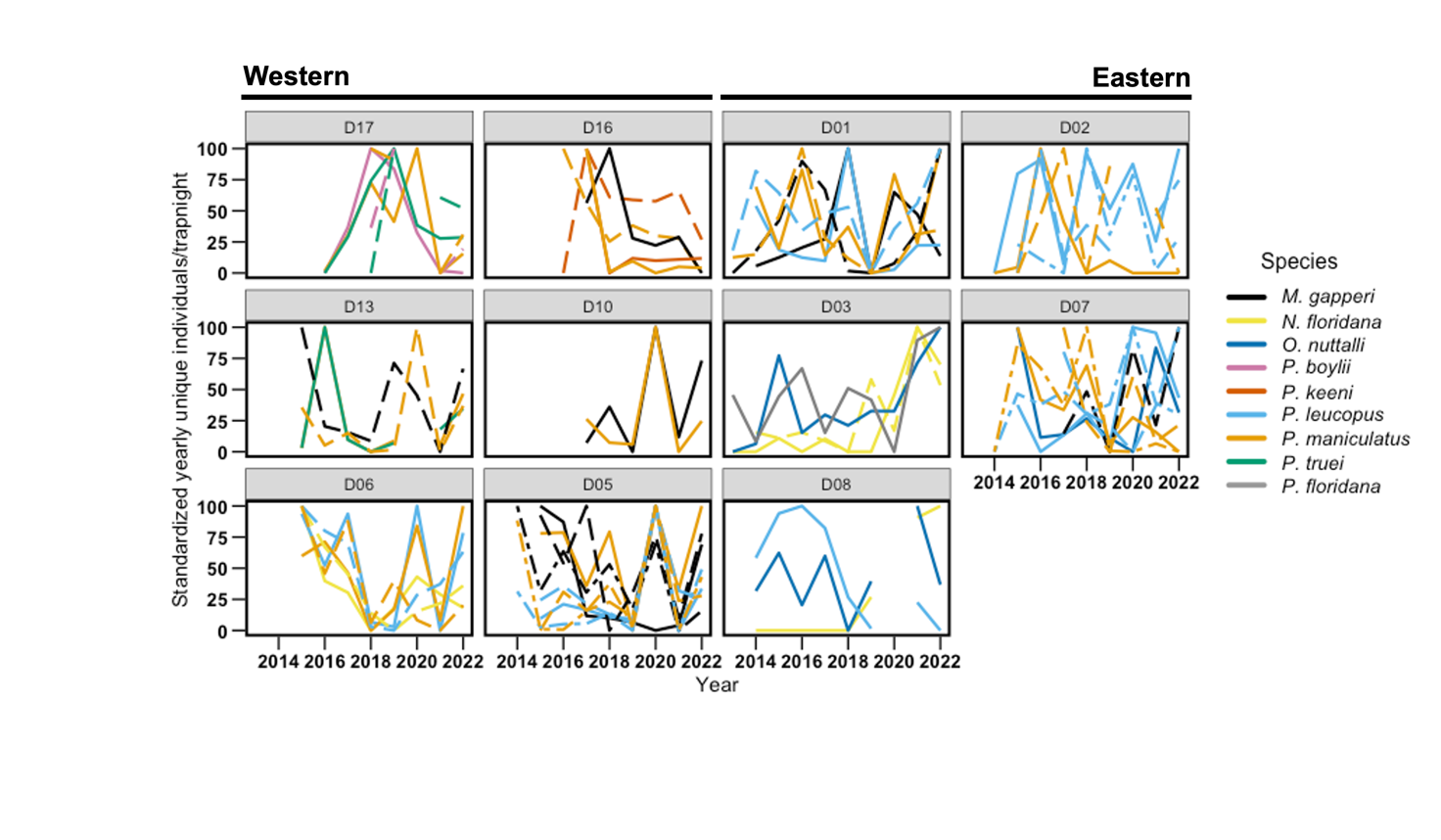


**Figure A2.** Temporal patterns of seed-eating small mammal populations within NEON domains, 2013-2022. Each line represents the standardized total yearly number of small mammals/trapnight for populations at each NEON site (standardized between 0-100), organized by domain. The line types (solid, dashed, or dash-dotted) represent different NEON sites within a given domain. Domains are separated geographically into roughly ‘Western’ or ‘Eastern’ for comparisons between near and far sites. Note that some data from 2020 is missing due to COVID-19 related constraints on staffing and field sampling. Values for yearly unique mammals per trap night were standardized between 0-100 across years prior to analysis to facilitate comparisons between sites and species with different baseline population sizes.


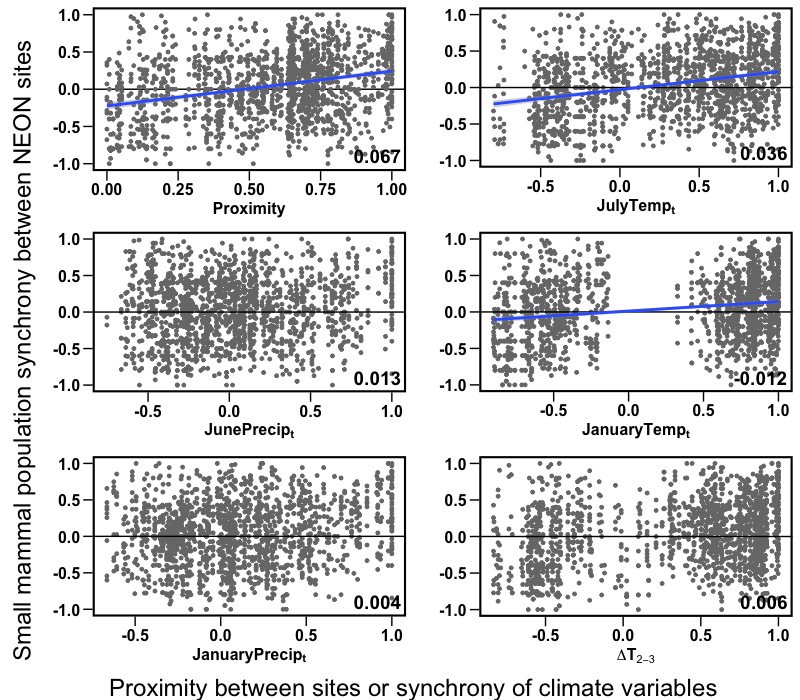


**Figure A3**. Relationships between the synchrony of mast-eating small mammal population dynamics and variables used in MRM analysis. Note that these figures represent direct associations between the response and predictor variables and show coefficients and relationships based on individual MRM models; these could vary from the partial MRM coefficients for these variables in the overall MRM analysis. Regression lines shown are for significant variables, and saturated MRM coefficients in the bottom right corner. Terms in the MRM included site proximity (ranging from 0-1, with 0 being the most distant NEON sites) and pairwise synchrony for a suite of climate variables. Each symbol represents a pair of species. Regression lines are shown with 95% confidence intervals.

**Appendix References**

Elias, S. P., J. W. Witham, and M. L. Hunter. 2004. *Peromyscus leucopus* Abundance and Acorn Mast: Population Fluctuation Patterns over 20 Years. Journal of Mammalogy 85:743–747.

Genoways, H. H., P. W. Freeman, and M. K. Clausen. 1997. Diet of a Relict Population of the Eastern Woodrat in Nebraska.

Hanley, T. A., and J. C. Barnard. 1999. Food resources and diet composition in riparian and upland habitats for Sitka mice, *Peromyscus keeni sitkensis*. Canadian Field Naturalist 133:401–407.

Hoffmeister, D. F. 1951. A Taxonomic and Evolutionary Study of the Piñon Mouse, *Peromyscus truei*. Fourth edition. The University of Illinois Press, Champaigne,Illinois,USA.

Hoffmeister, D. F. 1981. *Peromyscus truei*. Mammalian Species:1–5.

Jameson, E. W. 1952. Food of Deer Mice, *Peromyscus maniculatus* and *P. boylei*, in the Northern Sierra Nevada, California. Journal of Mammalogy 33:50.

Jones, C. A., and J. N. Layne. 1993. *Podomys floridanus*. Mammalian Species:1–5.

Krauss, R. 2018a. TOS Site Characterization Report: Domain 16.

Krauss, R. 2018b. TOS Site Characterization Report: Domain 01.

Krauss, R. 2018c. TOS Site Characterization Report: Domain 02.

Krauss, R. 2018d. TOS Site Characterization Report: Domain 07.

Krauss, R. 2018e. TOS Site Characterization Report: Domain 03.

Krauss, R. 2018f. TOS Site Characterization Report: Domain 06.

Krauss, R. 2018g. TOS Site Characterization Report: Domain 13.

Krauss, R. 2018h. TOS Site Characterization Report: Domain 10.

Krauss, R. 2018i. TOS Site Characterization Report: Domain 17.

Krauss, R. 2018j. TOS Site Characterization Report: Domain 05.

Krauss, R. 2018k. TOS Site Characterization Report: Domain 08.

Merritt, J. F., and J. M. Merritt. 1978. Population Ecology and Energy Relationships of *Clethrionomys gapperi* in a Colorado Subalpine Forest. Journal of Mammalogy 59:576–598.

Morzillo, A. T., G. A. Feldhamer, and M. C. Nicholson. 2003. Home range and nest use of the golden mouse (*Ochrotomys nuttalli*) in southern Illinois. Journal of Mammalogy 84:553–560.

NEON (National Ecological Observatory Network). 2023. Small mammal box trapping (DP1.10072.001), RELEASE-2023.

Thornton, M. M., R. Shrestha, Y. Wei, P. E. Thornton, S. C. Kao, and B. E. Wilson. 2022. Daymet: Monthly Climate Summaries on a 1-km Grid for North America.

U.S. Forest Service. 2021. Nuts. https://www.fs.usda.gov/wildflowers/ethnobotany/food/nuts.shtml.
